# Supplementary material for: Sexual selection and inbreeding: Two efficient ways to limit the accumulation of deleterious mutations
Source: Evol Lett. 2018 Dec 10;3(1):80–92. doi: 10.1002/evl3.93 (PMC6369961; doi:10.1002/evl3.93)
Supplement: Supplementary file 1 — Figure S1 [file EVL3-3-80-s001.pdf]

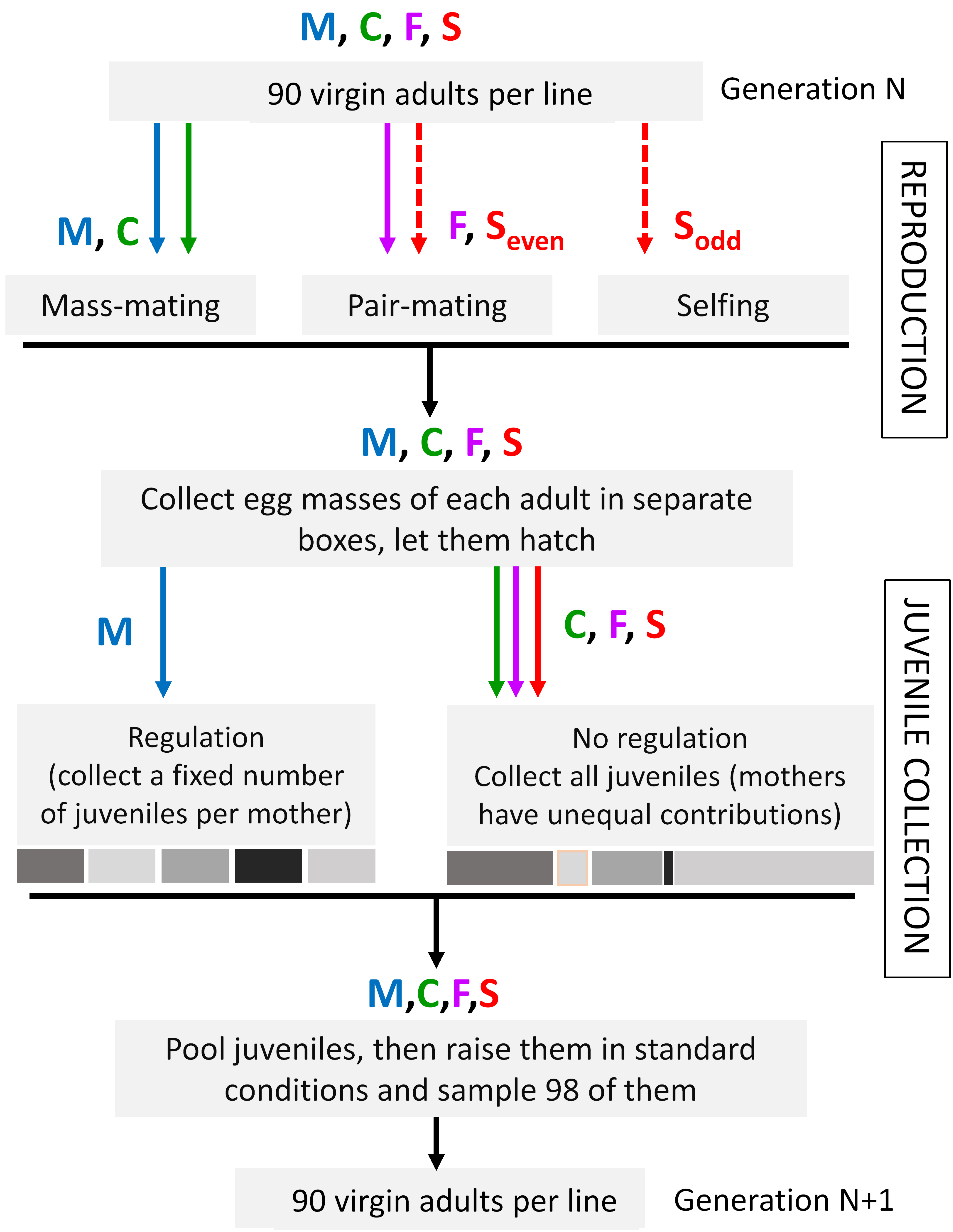
M, C, F, S
90 virgin adults per line
Generation N
REPRODUCTION
M, C
F, S_{even}
S_{odd}
Mass-mating
Pair-mating
Selfing
M, C, F, S
Collect egg masses of each adult in separate boxes, let them hatch
JUVENILE COLLECTION
M
C, F, S
Regulation (collect a fixed number of juveniles per mother)
No regulation Collect all juveniles (mothers have unequal contributions)
M,C,F,S
Pool juveniles, then raise them in standard conditions and sample 98 of them
90 virgin adults per line
Generation N+1
