# Supplementary material for: Sexual selection and inbreeding: Two efficient ways to limit the accumulation of deleterious mutations
Source: Evol Lett. 2018 Dec 10;3(1):80–92. doi: 10.1002/evl3.93 (PMC6369961; doi:10.1002/evl3.93)
Supplement: Supplementary file 2 — Table S1. Microsatellite variation in all experimental evolution lines (N = 32 individuals per line, 7 polymorphic loci) at the 49th generation. [file EVL3-3-80-s002.docx]

**Table S1.** Microsatellite variation in all experimental evolution lines (N=32 individuals per line, 7 polymorphic loci) at the 49^th^ generation. Each type (C, F, M and S) is represented by two replicate lines. Genetic diversity *H_e_*, observed heterozygosity *H_o_*, average number of alleles per locus *N_all_*, and inbreeding coefficient *f*.

|  | C1 | C2 | F1 | F2 | M1 | M2 | S1 | S2 |
| --- | --- | --- | --- | --- | --- | --- | --- | --- |
| *H_e_* | 0.526 | 0.219 | 0.272 | 0.321 | 0.366 | 0.381 | 0.141 | 0.163 |
| *H_o_* | 0.515 | 0.237 | 0.263 | 0.326 | 0.385 | 0.407 | 0.143 | 0.152 |
| *N_all_* | 3.714 | 1.714 | 1.857 | 2.429 | 2.571 | 2.429 | 1.571 | 1.571 |
| *f* | 0.022 | -0.080 | 0.034 | -0.014 | -0.055 | -0.071 | -0.012 | 0.070 |
